# Estimation of peak vertical velocity and relative load changes by subjective measures in weightlifting movements

**DOI:** 10.5114/biolsport.2022.106156

**Published:** 2021-08-27

**Authors:** Mark Chapman, Sam Damian Tomkins, Travis N Triplett, Eneko Larumbe-Zabala, Fernando Naclerio

**Affiliations:** 1 Institute for Lifecourse Development, School of Human Sciences, Centre for Exercise Activity and Rehabilitation, University of Greenwich, UK; 2 Department of Health and Exercise Science, Appalachian State University, Boone, NC, USA; 3 School of Doctorate and Research, European University of Madrid, Spain

**Keywords:** OMNI-RES (0–10) scale, Perceived Exertion, Hang Power Clean, Velocity-based training

## Abstract

To investigate the ability of the OMNI-RES (0–10) scale to estimate velocity and loading changes during sets to failure in the hang power clean (HPC) exercise. Eleven recreationally resistance-trained males (28.5 ± 3.5 years) with an average one-repetition maximum (1RM) value of 1.1 ± 0.07 kg body mass^-1^ in HPC, were assessed on five separate days with 48 hours of rest between sessions. After determining the 1RM value, participants performed four sets to self-determined failure with the following relative loading ranges: 60% < 70%, 70 < 80%, 80 < 90% and > 90%. The peak vertical velocity (PVV), and Rating of Perceived Exertion (RPE) were measured for every repetition of each set. The RPE expressed after the first repetition (RPE-1) and when the highest value of PVV was achieved during the set (RPE-max) were similar and significantly lower than the RPE associated with a 5% (RPE-5%) and 10% (RPE-10%) drop in PVV. In addition, the RPE produced at failure was similar to RPE-5% only for the heaviest range (≥ 90%). Furthermore, RPE-1 was useful to distinguish loading zones between the four assessed ranges (60 < 70%, vs. 70 < 80%, vs. 80 < 90%, vs. ≥ 90%). The RPE seems to be useful to identify PVV changes (maximal, 5% and 10% drop) during continuous sets to self-determined failure and to distinguish 10% loading zone increments, from 60 to 100% of 1RM in the HPC exercise.

## INTRODUCTION

The suitability of the perceptual response measured by rating of perceived exertion (RPE) scales to monitor changes in movement velocity during resistance exercises has been previously reported [1–3]. The ability to generate maximum muscular force to move heavy loads with a relatively high movement velocity and mechanical power production, as occurs in weightlifting movements, is limited by preserving a correct exercise technique and the capacity to attenuate selective fatigue of the fast motor units [4]. Monitoring the perceptual response during weightlifting exercises may represent a practical approach to estimate changes in both movement velocity [1] and the mechanical power output [5]. The use of subjective measurements can therefore help coaches to identify some technique related issues during weightlifting based workouts [5]. Few studies have examined the perceptual response during continuous sets of resistance exercises on a repetition-by-repetition basis [1, 2, 6, 7]. Chapman and colleagues [1, 2, 7] analyzed the ability of the perceptual response to reflect changes in movement velocity or to distinguish loading zones in squat and bench press during continuous sets to failure using a wide-load spectrum (30 to 100% of the 1RM). However, no study has examined the ability of RPE to mimic changes in the velocity of execution or to identify load increments in weightlifting type exercises [e.g., hang power clean (HPC)] that are currently used as effective conditioning component for several sports or fitness preparation [8, 9]. Sindiani et al. investigated the accuracy of predicting changes in movement velocity (as a percentage of the first repetition) in squat and bench press [6]. The actual changes in movement velocity were measured with a linear position transducer and reported as a percentage of actual velocity of the first repetition during sets of 8 repetitions performed at 60 or 70% of one repetition maximum (1RM). Overall, the participants underestimated the relative changes in movement velocity, and this error was gradually increased as the set progressed. The authors suggested that using the perceptual response to estimate relative changes in movement velocity may be better suited for sets of fewer repetitions (up to 5) and velocity-loss threshold of 5–10% [6].

**TABLE 1:** Mean (SD) of the total number of repetitions and PVV measured at the corresponding time points in each of the four assessed load ranges in the Hang Power Clean exercise.

Slot Ranges as % 1RM	Total Repetitions	PVV-1 (m^.^s^-1^)	PVV-Max (m^.^s^-1^)	PVV-5% (m^.^s^-1^)	PVV-10% (m^.^s^-1^)	PVV-F (m^.^s^-1^)
> 60–70	17.4 ± 4	2.0 ± 0.3	2.1 ± 0.3	1.9 ± 0.3	1.8 ± 0.3	1.7 ± 0.2
> 70–80	12.4 ± 2.5	1.8 ± 0.3	1.9 ± 0.3	1.8 ± 0.3	1.7 ± 0.2	1.6 ± 0.2
> 80–90	6.8 ± 0.7	2.0 ± 0.6	2.0 ± 0.6	1.9 ± 0.5	1.8 ± 0.5	1.7 ± 0.52
> 90–100	3.1 ± 1.0	1.7 ± 0.3	1.7 ± 0.3	1.6 ± 0.3	1.5 ± 0.3	1.6 ± 0.3

PVV: peak vertical velocity; PVV-1: peak vertical velocity achieved during the first repetitions; PVV-max: highest value of peak vertical velocity; PVV-5%: peak vertical velocity measured when a 5% decrease was determined PVV-10%: peak vertical velocity measured when a 10% decrease was determined; PVV-F: peak vertical velocity measured during the last completed repetition.

**TABLE 2:** Mean ± SD for the RPE and the five analyzed time points within the sets and across the four assessed ranges.

Variables	Percentage ranges				One-way ANOVA (4 assessments)
	60 to < 70%	70 to < 80%	80 to < 90%	≥ 90%	
RPE (0–10)	*	†	‡	j	
RPE-1	4.5 ± 0.7^a^	5.5 ± 0.7^a^	6.5 ± 0.8 ^a^	8.4 ± 0.7 ^a^	F(3,30)=89.00, p<0.001, $\eta_{G}^{2}$=0.81
RPE-max	5.3 ± 1.3^b^	5.9 ± 0.8 ^c^	6.7 ± 0.9^c^	8.4 ± 0.7	F(3,30)=22.81, p<0.001, $\eta_{G}^{2}$=0.61
RPE-5%	6.1 ± 1.6^d^	7.4 ± 1.2^d^	8.0 ± 1.1	9.0 ± 0.4	F(3,30)=130.82, p<0.001, $\eta_{G}^{2}$=0.87
RPE-10%	8.5 ± 1.0	9.1 ± 0.7	8.5 ± 1.2	9.4 ± 1.2	F(3,30)=2.57, p<0.073, $\eta_{G}^{2}$=0.15
RPE-F	9.9 ± 0.3	9.5 ± 0.5	9.5 ± 0.5	9.4 ± 0.5	F(3,30)=2.59, p<0.072, $\eta_{G}^{2}$=0.14

One-way ANOVA (5 time points)	F(4,40)=69.97, p<0.001, $\eta_{G}^{2}$ =0.79	F(4,40)=60.34, p<0.001, $\eta_{G}^{2}$=0.81	F(4,40)=34.75, p<0.001, $\eta_{G}^{2}$=0.60	F(4,40)=12.77, p<0.001, $\eta_{G}^{2}$=0.41	

RPE: Rating of perceived exertion from OMNI-RES (0–10) scale; RPE-1: RPE value expressed after the first repetitions; RPE-max: RPE expressed after performing the repetition that produced the highest peak vertical velocity; RPE-5%: RPE value measured when a 5% drop in peak vertical velocity was determined; RPE-10%: RPE value measured when a 10% drop in peak vertical velocity was determined RPE-F: RPE value expressed immediately after completed the set.

Differences within ranges: *p < 0.05 between the five time points with the exception of RPE-1 vs. RPE-max (p = 1.000); † p < 0.05 between the five time points with the exception of RPE-1 vs. RPE-max (p = 0.531) and RPE-10% vs. RPE-F (p = 0.162); ‡ p < 0.01 between the five time points with the exception of RPE-1 vs. RPE-max (p = 1.000), RPE-5% vs. RPE-10% (p = 0.519) and RPE-10% vs. RPE-F (p = 0.162); j p < 0.05 between RPE-1 vs. RPE-5%, RPE-10% and RPE-F, RPE-max vs. RPE-10% and RPE-F.

Differences across ranges: ^a^ p < 0.001 between all the four assessed ranges, ^b^ p < 0.001 from 60 < 70 to 80 < 90 and ≥ 90%, ^c^ p < 0.05 from 70 < 80% and 80 < 90% to ≥ 90%; ^d^ p < 0.05 from 60 < 70% to all the other ranges and from 70 < 80% to all the other ranges.

**TABLE 3:** Mean CI (95%) determined on the RPE main variables determined along the four-repetition to failure test.

1 RM ranges	RPE-1/RPE max		RPE-5%		RPE-10%		RPE-F	
	Lower	Upper	Lower	Upper	Lower	Upper	Lower	Upper
60 < 70%	4	5	5	7	8	9	> 9	10
70 < 80%	5	6	7	8	8	> 9	> 9	10
80 < 90%	6	7	8	> 8	9	> 9	> 9	10
≥ 90%	8	9	> 8	> 9	9	> 9	> 9	10

RPE-1: OMNI-RES scale value determined after doing the first repetition of each repetition to failure test. RPE-max: OMNI-RES scale value of the repetition where the peak vertical velocity was reached in each repetition to failure set. RPE-5%: OMNI-RES scale value expressed when a 5% decrease in the peak vertical velocity was determined along each repetition to failure set RPE-10%: OMNI-RES scale value expressed when a 10% decrease in the peak vertical velocity was determined along each repetition to failure set. RPE-F: OMNI-RES scale value expressed after performing the last repetition of each ach repetition to failure set.

The sensitivity of the perceived exertion to differentiate specific events within a set where the peak vertical velocity (PVV) of the bar peaks, drops below a certain level (e.g., 10%) from the maximum, or where the athlete approaches muscular failure still needs specific consideration when performing weightlifting movements within a workout routine. Success in weightlifting exercises has been associated with the level of PVV [10]. The highest PVV value occurs during the second pull (clean or snatch exercises) after the bar passes over the knee reaching the mid-thigh position by a triple extension of the hip, knee and ankle joints [8]. Consequently, the aim of the present study was twofold: (i) to examine the ability of the OMNI-RES (0–10) scale to estimate changes in movement velocity (e.g., instances where velocity peaks, decreases by 5% and 10% from the maximum or the set terminates due to muscle failure) using moderate to heavy loads in the HPC exercise; (ii) to investigate the ability of the RPE to discriminate between relative loads across a wide range, from 60 to 100% of 1RM, divided into 10% incremental slots. Our hypotheses are: (i) the RPE expressed at the end of each repetition will show differences between specific moments within the set where the velocity concomitantly decreases as the set approaches muscular failure; and (ii) the RPE measured at the beginning of each set will differentiate relative loads (as a percentage of 1RM) utilized.

## MATERIALS AND METHODS

### Participants

Eleven volunteers, recreationally resistance-trained males (age 28.5 ± 3.5 years, body mass 82.9 ± 7.6 kg, and height 177.4 ± 7.2 cm) took part in this study. To be eligible, participants had to be free of injury in the last three months before the intervention. They were furthermore required to have a minimum of 2 years of experience with resistance training at a frequency of 2 to 3 times per week. Additionally, they had to be performing weightlifting exercises (e.g., HPC) as a part of their regular routine for a minimum of 6 months. Given the relevance of the level of performance on enhancing technique and reaching higher PVV values in weightlifting exercises performed with similar relative loads [11], to maintain a homogenous sample, only recreationally-trained individuals with no regular participation in bodybuilding, powerlifting or weightlifting were recruited. Additionally, participants were requested not to ingest ergogenic aids, and stick to a normal dietary habit. If they deviated from this recommendation, their data were excluded from the analysis.

The sample size was determined using G* Power 3.1 software and estimated based on RPE scores reported in previous studies using similar designs and population [1, 2]. Assuming an α-error of 0.05, coefficient of 0.5, nonsphericity correction of 1, and an effect size *f* = 0.64, a minimum sample size of n = 5 was estimated to achieve an 80% statistical power.

### Procedures

Two days of familiarization were conducted with participants before the study commenced. These sessions aimed to ensure (i) all participants were skilled enough to perform the HPC exercise and (ii) participants understood the 0–10 points OMNI-Resistance Exercise Scale (OMNI-RES) procedures to properly disclose the RPE score for the whole body. Participants then performed a standardized warm-up consisting of dynamic stretching and calisthenics exercises, followed by 10 to 15 weightlifting movements performed with an empty barbell. Thereafter, in order to optimize the neuromuscular preparation a specific warm-up phase composed of 1 repetition using 60%, 70%, 80% and 90% of 1RM separated by 2 min rest was implemented.

**FIG. 1:**
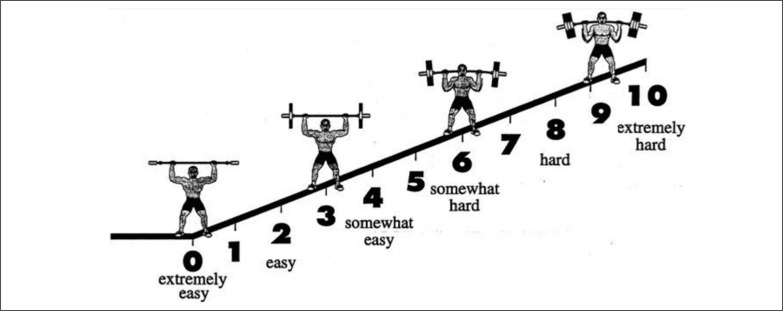
The 0–10 points OMNI-Resistance Exercise Scale (OMNI-RES).

The OMNI-RES (0–10) scale includes both verbal and mode-specific pictorial descriptors distributed along a comparatively narrow response range of 0 to 10 (Figure 1). These characteristics make this scale a useful tool to estimate effort during weightlifting exercises [12].

### Exercise

The HPC was performed using free weights on a weightlifting platform according to the technique described elsewhere [13, 14]. Briefly, participants initiated the movement from the standing position holding the barbell with a slightly wider-than-shoulder width grip and lowered the bar to midthigh position, and then lifted the bar upward applying the maximum possible impulse until receiving the barbell on the shoulders at or slightly above parallel-squat position with both elbows pointing forward [15]. All participants were instructed to push the bar away or simply drop it on the platform [15] if they felt that they were going to miss the lift or not maintain control of the bar. For safety reasons, the surrounding area of the platform was always cleared of other persons and equipment. Furthermore, all participants performing the lifts wore appropriate sports clothes including footwear for lifting exercises. Feedback from a qualified instructor [certified strength and conditioning coach (CSCS)] ensured that athletes used proper HPC technique.

### Assessments

#### 1RM and Repetitions to Self-determined Momentary Failure Tests (RTFT)

The 1RM HPC was determined in the first session according to the procedures described by McGuigan [16]. After 48-h rest and based on the 1RM results, participants performed four assessment sessions separated by 48 hours of rest. Each session comprised of only one repetition to RTFT using the following 1RM range percentages: 60 < 70%; 70 < 80%; 80 < 90% and ≥ 90%. Self-determined momentary failure was defined as the endpoint of the set where participants felt they cannot complete the lift using a proper technique [17].

As the availability of the free weight equipment (20 kg Olympic bar, 1.25, 2.5, 5, 10, 15 and 20 kg discs) did not always allow for an exact match to each calculated relative load (e.g., 60%), the nearest load (kg) to the inferior limit in each of the 4 assessed ranges was utilized for the test. To minimize the accumulated fatigue effect, sequencing of the RTFT was randomized. Furthermore, participants were asked to abstain from any unaccustomed or hard exercise and refrain from caffeine intake, whilst maintaining similar sleeping hours and daily activities during the testing period. All assessments were performed during the afternoon (12:00 to 6:00 pm). In the case of habitual caffeine users, to avoid privation syndrome, they were advised to progressively decrease caffeine intake 1 week before the study began [18].

#### Measurement of peak vertical velocity

A validated [19] optoelectronic infrared camera system (Velowin, Deportec, Spain) with a fixed sampling frequency of 500 Hz was used to track a retroreflective strip placed at the center of the bar during the HPC execution. The device was connected to a computer through a USB interface and the proprietary software (Velowin 1.6.314). Numeric and graphical real-time information after each repetition was obtained. All data were filtered using a low pass 10 Hz cut-off filter before calculating the displacement of the bar and the PVV.

The analysis of the PVV achieved during the RTF test was based on five specific events determined at: (i) the first repetition (PVV-1); (ii) the repetition where the maximum value of PVV was achieved along the corresponding set (PVV-max); (iii) the repetition where a drop of 5% in the PVV from the PVV-max was identified (PVV-5%); (iv) the repetition where a drop of 10% in the PVV from the PVV-max was identified (PVV-10%) and (v) the PVV measured during the last successfully completed repetition (PVV-F) of each set. The two velocity thresholds (PVV-5% and PVV-10%) were determined based on practical recommendations for athletes using weightlifting exercises where a small drop of velocity (≤ 10%) is associated with an undesired reduction of the power output and poor performance [20, 21]. The criterion to determine the critical point associated with both the PVV-5% and PVV-10 was the execution of two continuous repetitions with a 5% and 10% reduction from the PVV-max, respectively. Furthermore, in order to determine the total drop in movement velocity compared with the initial and the maximal velocity achieved within each set, the PVV-F was also considered.

#### Control of the rating of perceived exertion (RPE)

During the RTFT the participants were instructed to verbally report the RPE value indicating a number of the OMNI-RES (0–10) scale (Figure 1) that reflected their overall whole-body effort [22] immediately after the completion of every repetition of the HPC while holding the bar at the mid-thigh position. A minimal break (~1 sec) between repetitions was allowed for the participants to report their RPE scores. Before starting each RTFT, the investigators provided the same simplified instruction to the participants: “after the completion of each repetition, you need to verbally report how much effort have you experienced during the exercise”. A rating of 10 was considered to be the perceived maximum effort to perform the specific assessed exercise [23] and associated with the most stressful effort ever performed [24]. Conversely, a rating of 0 was associated with no effort (seated or resting), and a rating of 1 corresponded to the perception of effort while performing an extremely easy physical task [25]. A large (A3 size 29.7 x 42 cm) figure showing the OMNI–RES (0–10) scale was always placed on the wall (less than 2 m) in front of the participants during the assessments.

#### Dependent Variable

The ability of the perceptual response to reflect changes in PVV was assessed by the RPE scores measured at the previously identified five PVV critical points (PVV-1, PVV-max, PVV-5%, PVV-10% and PVV-F): (i) RPE-1: OMNI-RES value of the first repetitions of each corresponding set, (ii) RPE-max: OMNI-RES value measured where the highest value of PVV was measured for each corresponding set, (iii) RPE-5%: OMNI-RES value measured when a 5% drop in PVV was determined during the corresponding set (iv) RPE-10%: OMNI-RES value measured when a 10% drop in PVV was determined during corresponding set and (v) RPE-F OMNI-RES value measured immediately after the end of the last repetition of each corresponding set.

The test-retest reliability coefficients (95% ICCs) and standard error of measurement (SEM) for the five RTF tests were above 0.92 and between 0.13 to 0.02 m^.^s^-1^ or 0 to 1.8 m^.^s^-1^ considering the five time points measured at the PVV and the OMNI-RES (0–10) scale, respectively.

### Statistical Analyses

Means and standard deviations (SD) were determined for all variables analyzed during the 1RM and RTF tests. The Shapiro-Francia test was applied to assess normality. To analyze the existence of differences within a continuous set between the RPE values measured at the five critical points identified within each RTF set, one-way repeated measures analysis of variance (ANOVA) was applied for each of the four tested load ranges (first hypothesis). Repeated measures ANOVAs were performed to examine differences between the five perceptual events (RPE-1, RPE-max; RPE-5%; RPE-10% and RPE-F) across the four loading ranges, (second hypothesis). Bonferroni-adjusted pairwise comparisons were conducted when appropriate. Generalized eta squared ($\eta_{G}^{2}$) and Cohen´s *d* values were reported to provide an estimate of standardized effect size (small d = 0.2, $\eta_{G}^{2}$ = 0.01; moderate d = 0.5, $\eta_{G}^{2}$ = 0.06; and large d = 0.8, $\eta_{G}^{2}$ = 0.14). The significance level was set at 0.05. Data analyses were performed with JASP 0.12 (University of Amsterdam).

### Ethics

All experimental procedures were conducted following the Declaration of Helsinki and approved by the University Research Ethics Committee. All participants were informed of the benefits and risks of the investigation before signing the informed consent to participate in the study.

## RESULTS

The HPC 1RM mean value was 89.1 ± 8.8 kg (1.1 ± 0.07 kg^.^ body mass^-1^). Table 1 describes the total number of repetitions and PVV values measured across ranges at the corresponding identified five time points in each of the RTF tests.

Table 2 shows the mean ± SD of the RPE values, and the corresponding five time points analyzed along the RTFT within and across the four assessed ranges.

### Comparison of the five critical points within each range

Main time effects were observed for the four ranges. Significant differences and large effect sizes (d > 0.80) were determined by comparing the five critical points within the four assessed load ranges. Nonetheless, the following exception showed no significant differences (p > 0.05) (i) for all the ranges RPE-1 was similar to RPE-max; (ii) at 70 < 80%, 80 < 90% and ≥ 90% RPE-10% was similar to RPE-F (iii) at 80 < 90% and ≥ 90%, RPE-5% was similar to RPE-10% (iv) ≥ 90% RPE-max was similar to RPE-5% and RPE-5% was similar to RPE-F.

### Comparison between critical points across ranges

Significant main range effects were observed for the RPE-1; RPE-max and RPE-5% but not for the RPE-10% and RPE-F. A Pairwise comparison revealed significant differences and large effect sizes (d > 0.80) between the RPE-1 expressed in each of the four assessed ranges. The RPE-max was lower at 60 < 70% compared to that measured at 80 < 90% and ≥ 90%. Furthermore, the RPE-max at 70 < 80% and 80 < 90% was lower than the observed at ≥ 90%. Finally, the RPE-5% measured at 60 < 70% was lower than the other three ranges and the RPE-5% at 70 < 80% was lower to the values expressed at the two highest ranges. It is worth noticing that the RPE-F was similar between ranges.

Table 3 depicts the 95% CI limits for the analyzed RPE variables. As RPE-1 and RPE-max produced very similar values, their scores are merged. For practical uses, the values have been rounded using the nearest lower unit. When the resulted scores were in the middle, we opted to recommend an RPE-score higher than the referenced value (e.g., > 5) but still lower than the next-superior unit (e.g., 6). The RPE-1 indicates 10% load increases and may help athletes to differentiate loading zones. The RPE-5% and RPE-10% reflect changes in the PVV while performing continuous sets in the HPC exercise.

## DISCUSSION

The main findings from the present investigation using the PVV as the referential variable to explore whether the RPE reflects load increments and velocity changes when performing continuous sets of the HPC exercise were as follows: (i) rating the perceptual response on a repetition by repetition basis is a valuable methodology to detect changes in the PVV during continuous sets until self-determined momentary failure; and (ii) the initial RPE is useful to indicate increases of the loading zone over 10% incremental ranges (from 60 to > 90% 1RM) in the HPC exercise.

Using a similar criterion as in previous investigations analyzing resistance exercises (e.g., bench press [1] or squat [2]), five crucial events were analyzed: (i) the initial RPE-1 that was associated with the relative load used (ii) the perception expressed when the highest value of PVV is achieved (RPE-max), (iii) the perception expressed at a drop of 5% of the PVV (RPE-5%), (iv) the perception expressed at a drop of 10% of the PVV (RPE-10%), and (v) the perception associated with the self-determined momentary failure (RPE-F). The RPE-1 and RPE-max were similar in all the four assessed ranges. Additionally, both (RPE-1 and RPE-max) were different from other critical points (RPE-5%, RPE-10% and the RPE-F), being only similar to RPE-5% for the heaviest (≥ 90%) loading range. The observed results support the use of the perceptual response measured at the end of each repetition during continuous sets in weightlifting movements to estimate the moment where the PVV drops -5%, -10% or the athlete is approaching self-determined momentary failure.

Collectively, the RPE score collected on a repetition-by-repetition basis emerges as a practical alternative to traditional criteria such as the percentage of 1RM or the repetition maximum continuum for distinguishing increments of the loading zone and also to terminate the set when certain changes in the movement velocity or proximity to muscular failure are identified. Additionally, within the context of the limitation of RPE scales [23] the appropriate use of the perceptual response could apply under similar criteria as the velocity control training [26, 27]. This method advocates for the determination of velocity thresholds considering the gradual decrease in the movement velocity that occurs as the sets or workout progress. To illustrate, instead of prescribing a given number of repetitions per set, an open-ended number of repetitions is indicated with each set terminated when the movement velocity drops below a given threshold, i.e. 10%, 20% or 30% of the maximal estimated or measured at the beginning of the workout [27, 28]. In line with the previous rationale the RPE scores expressed over an ongoing set can be associated with pre-established perceptual threshold scores to stop the set when the RPE reaches an undesired value, for example, 8 (~10% PVV loss) when exercising at 60 < 70% of 1RM in the HCP. The use of the perceptual response for volume autoregulation was proposed in powerlifting [29]. An initial set performed with a specific range of repetitions corresponding to the proposed training outcome and a target RPE score is proposed, e.g., 3 repetitions, RPE 9; 2 repetitions, RPE 8, or 8 repetitions, RPE 8 for increasing strength, power output or hypertrophy, respectively. Once the reference load eliciting the target RPE score is identified, the subsequent sets are conducted with a reduced load (~2 to 6% less) but maintaining the same number of prescribed repetitions until the target RPE is perceived again and consequently dictating the termination of the sets [29]. Accordingly, the perception of effort rated after each repetition can be used to identify the loading zone by the initial RPE (RPE-1) and to establish RPE threshold scores. For instance, when the pre-established non-desired perceptual scores are reached, the set is stopped to reconfigure (e.g., extend the rest period, change the load or number of repetitions) or even end the workout.

Previous studies using jumps [30] or resistance [28] but not weightlifting type exercises reported higher metabolic stress and greater hypertrophic related outcomes after the completion of sets with larger velocity losses (e.g. > 10%) [30]. Conversely, smaller losses (e.g., < 10%), maintaining ≥ 90% of the maximal velocity, were more effective to enhance mechanical power output. To the best of the authors’ knowledge, this is the first study in analyzing the use of the perceptual response to estimate small losses of PPVs during one weightlifting exercise. Exercising above the used velocity thresholds (-5, and -10%) could be non-desirable when training for mechanical power [21, 31]. Indeed, the PVV-10% was very similar to PVV-F (Table 1). Given the similar scores produced by the RPE-10% and RPE-F, it is possible to infer that a 10% loss in PVV indicates an athlete is approaching failure.

In summary, the analysis within each range permits the acceptance of the first hypothesis supporting the ability of the RPE to monitor changes in the PVV during continuous sets in the HPC exercise. The analysis across the ranges indicates that RPE-1 represents a useful reference to discriminate between 10% increments of 1RM when exercising with moderate (> 60% 1RM) to maximal (≥ 90%) loads. Either the RPE-1 or the RPE-max are different when compared between the four analyzed ranges (60% to ≥ 90% of 1RM). Therefore, we accept the second hypothesis of utilizing the RPE to differentiate loading ranges based on 10% slot of 1RM percentages in the HPC exercise.

The present study supports the use of the OMNI-RES (0–10) scale to identify loading zones and to estimate changes in the PVV occurring at different instances along with a continuous set with moderate to heavy loads in the HPC exercise. For example, at the beginning of the workout an athlete can estimate the relative load based on the RPE expressed after the first repetition (RPE-1): ~4 to 5 for 60 < 70% 1RM, 5 to 6 for 70 < 80% 1RM, 6 to 7 for 80 < 90% and 8 to ~9 when using > 90% 1RM. Athletes will be instructed to perform the exercise with a maximal possible impulse considering RPE values ~5, 7, 8 or > 8 using 60 < 70%, 70 < 80%, 80 < 90% or > 90% 1RM, respectively indicate PVV loss of ~5% (Table 3). Furthermore, RPE scores ~8 (for 60 < 70% and 70 < 80%) and 9 (for 80 < 90% or > 90%) are associated with PVV drops of ~10% suggesting potential technique disruptions with a concomitant proximity to failure.

Although velocity control devices such as the optoelectronic system used in this study or others, e.g., accelerometers, linear transducers, or even iPhone app are currently used to measure changes in movement velocity when performing resistance [32] and weightlifting exercises [12], from a practical point of view the availability of these devices could be difficult to implement on a day-by-day basis. Consequently, athletes and coaches would acknowledge alternative indirect methods, although not extremely accurate, can provide valuable support to monitor performance progression during resistance training.

## CONCLUSIONS

Our results corroborate the use of the RPE measured from the OMNI-RES (0–10) scale to identify PVV changes (maximal, 5% and 10% drop) during continuous sets to self-determined momentary muscular failure and to distinguish 10% loading-range increments, from 60 to 100% of 1RM in the HPC.
